# Thyroid-Associated Ophthalmopathy after Radioactive Iodine Therapy for Metastatic Follicular Thyroid Carcinoma

**DOI:** 10.1155/2021/3024639

**Published:** 2021-06-11

**Authors:** Daisuke Murayama, Soji Toda, Yoichiro Okubo, Hiroyuki Hayashi, Ai Matsui, Mio Yasukawa, Hiroyuki Iwasaki

**Affiliations:** ^1^Department of Breast and Endocrine Surgery, Kanagawa Cancer Center, 2-3-2 Nakao Asahi-ku, Yokohama, Kanagawa 241-8515, Japan; ^2^Department of Pathology, Kanagawa Cancer Center, 2-3-2 Nakao Asahi-ku, Yokohama, Kanagawa 241-8515, Japan; ^3^Department of Pathology, Yokohama Municipal Citizen's Hospital, 1-1 Mitsuzawanishicho Kanagawa-ku, Yokohama, Kanagawa 221-0855, Japan

## Abstract

Thyroid-associated ophthalmopathy (TAO) is an inflammation of the extraocular muscles and periorbital connective tissue caused by autoantibodies against common antigens to both the thyroid and orbit. The release of antigens and induction of hypothyroidism caused by radioactive iodine (RAI) therapy may exacerbate TAO. Here, we present the case of a 67-year-old-woman treated with RAI therapy for metastatic follicular thyroid carcinoma who presented with TAO during the course of sorafenib administration. Tg and TgAb levels were gradually decreased with sorafenib and lenvatinib treatment, and TAO was improved without any ophthalmologic treatment.

## 1. Introduction

Thyroid-associated ophthalmopathy (TAO) is an inflammation of the extraocular muscles and periorbital connective tissue caused by autoantibodies against common antigens to both the thyroid and orbit such as thyroid-stimulating hormone receptor (TSH-R) [1]. Several reports suggest that the release of antigens and induction of hypothyroidism caused by radioactive iodine (RAI) therapy may exacerbate TAO [2]. Sorafenib is an orally administered inhibitor of vascular endothelial growth factor receptor- (VEGFR-) 1, 2, and 3, RET, RAF, and platelet-derived growth factor receptor (PDGFR)-*β* [3]. Lenvatinib is also an orally administered inhibitor of VEGFR-1, -2, and -3, fibroblast growth factor receptor-1 to -4, PDGFR-*α*, RET, and KIT [4]. TAO after RAI for thyroid carcinoma after total thyroidectomy has been reported in a few studies [5–7]; however, the improvement in TAO during the course of sorafenib and lenvatinib treatment for metastases of RAI-refractory differentiated thyroid carcinoma after total thyroidectomy has not been reported.

## 2. Case Presentation

**Figure 1:**
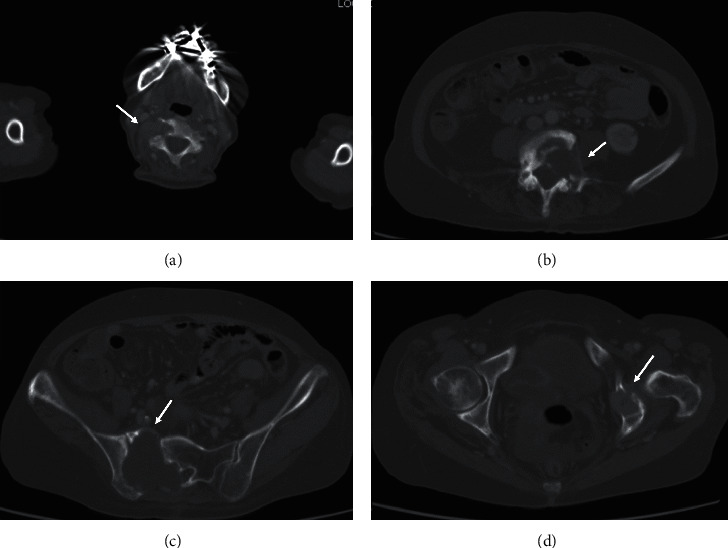
Computed tomography revealing bone metastasis (arrow). (a) C5, (b) L4, (c) S1, and (d) left acetabular cartilage.

**Figure 2:**
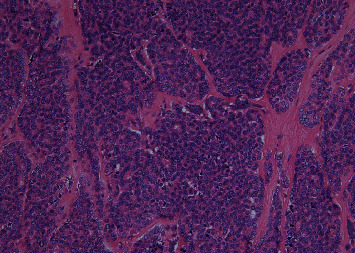
Pathological diagnosis of poorly differentiated carcinoma derived from follicular thyroid carcinoma with minimal invasion; Victoria Blue hematoxylin and eosin staining, 200x.

**Figure 3:**
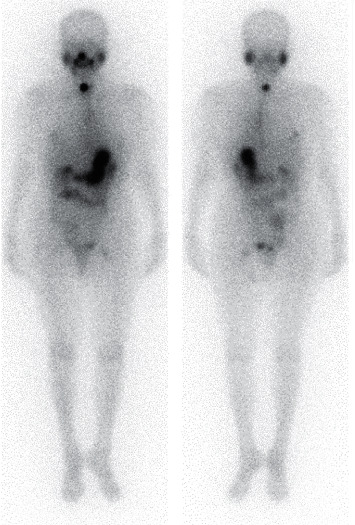
Iodine-131 scintigraphy after radioactive iodine therapy. No uptake was observed in the metastatic lesions.

**Figure 4:**
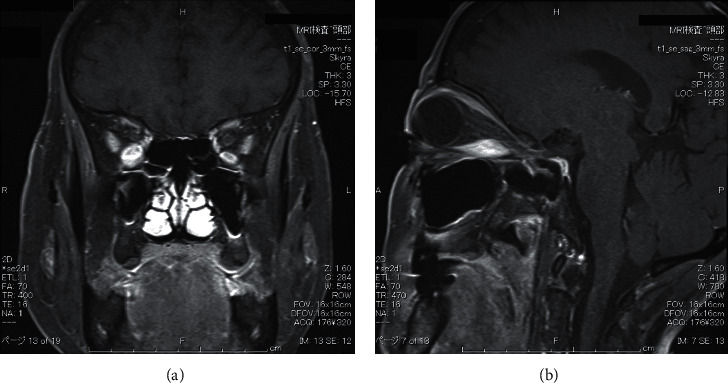
Magnetic resonance imaging revealing thickening of the right inferior rectus muscle (asterisk).

**Figure 5:**
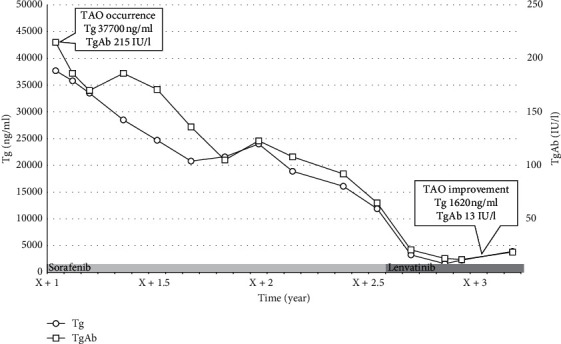
Change of thyroglobulin (Tg) and thyroglobulin antibody (TgAb) levels (from thyroid-associated ophthalmopathy occurrence to improvement).

A 67-year-old-woman who presented with back pain was referred to our institution to evaluate the cause. Computed tomography (CT) revealed a thyroid tumor and multiple vertebral bone metastases (C5, L4, S1, and left acetabular cartilage) (Figure 1). Biopsy of L4 was performed, and pathological findings showed metastasis of follicular thyroid carcinoma. Denosumab was initiated to address multiple bone metastases, and the patient underwent total thyroidectomy and central neck dissection. Pathological diagnosis was poorly differentiated carcinoma derived from follicular thyroid carcinoma with minimal invasion (Figure 2). After the surgery, the patient received TSH-suppressive therapy with levothyroxine sodium hydrate 100 *μ*g daily and treated with RAI therapy (3.7 GBq). However, no uptake was observed in the metastatic lesions (Figure 3). Three months after RAI therapy, external beam radiation therapy (36 Gy/12F) was performed to the lumbar spine and sacrum. Six months after RAI, sorafenib 800 mg was initiated with gradually decreasing dosage because of diarrhea. One year after RAI, the patient presented with diplopia, and magnetic resonance imaging revealed a thickening of the right inferior rectus muscle (Figure 4). Laboratory data obtained the following findings: thyrotropin receptor antibody (TRAb) 11.8 IU/L, euthyroid, thyroglobulin (Tg) 37700 ng/mL, and thyroglobulin antibody (TgAb) 215 IU/L (the normal value of TRAb, Tg, and TgAb are 2.0 IU/L or less, 33.7 ng/mL or less, and 28.0 IU/mL or less, respectively). Lumbar puncture and cytology found no abnormalities, and the patient was diagnosed with TAO. Despite the recommendation of an ophthalmologist, she refused treatment for TAO. However, Tg and TgAb gradually decreased with sorafenib and lenvatinib administration, and TAO was improved two years after occurrence (Figure 5). At the time of TAO improvement, Tg and TgAb levels were 1620 ng/mL and 13 IU/L, respectively. During the course of treatment, no changes in bone metastasis were observed on CT.

## 3. Discussion

In 1967, Kriss et al. [8] initially reported the exacerbation of TAO after RAI therapy. Radiation injury appears to induce thyroid antigen leakage, leading to an increased production of TSH-R antibodies [9], which may cause orbital injury, since TSH-R is expressed in orbital tissue [10]. TAO may develop at any time 1–24 months after RAI for Grave's disease [11]. However, Bartalena and Tanda [12] reported that TAO may occur in patients with no thyroid dysfunction.

Only three case reports of TAO after RAI for thyroid carcinoma have been reported [5–7], and all were treated with an ablative dose (2.2 to 3.7 GBq). Despite the absence of thyroid tissue after total thyroidectomy for nonmetastatic thyroid carcinoma, TAO occurred after RAI [6, 7]. The time period from RAI to TAO onset is 3–34 years [5–7]. All three previous cases demonstrated no thyroid dysfunction, as with our case.

Lahooti et al. [13] reported a significant positive correlation between serum Tg levels and the presence and severity of ophthalmopathy in patients with Graves' disease. As for our case, the decline of thyroid antigen (Tg and TgAb) with sorafenib and lenvatinib administration resulted in TAO improvement (Figure 5). However, TRAb was evaluated only once at TAO onset, and we could not establish whether TRAb was improved with sorafenib and lenvatinib treatment.

In conclusion, our case presented with TAO after RAI. Tg and TgAb levels were gradually decreased with sorafenib and lenvatinib treatment, and TAO was improved without any ophthalmologic treatment.

## Data Availability

The datasets used during the current study are available from the corresponding author upon request.
